# Challenges and approaches of single-crystal Ni-rich layered cathodes in lithium batteries

**DOI:** 10.1093/nsr/nwad252

**Published:** 2023-09-22

**Authors:** Jiangtao Hu, Hongbin Wang, Biwei Xiao, Pei Liu, Tao Huang, Yongliang Li, Xiangzhong Ren, Qianling Zhang, Jianhong Liu, Xiaoping Ouyang, Xueliang Sun

**Affiliations:** Graphene Composite Research Center, College of Chemistry and Environmental Engineering, Shenzhen University, Shenzhen518060, China; Graphene Composite Research Center, College of Chemistry and Environmental Engineering, Shenzhen University, Shenzhen518060, China; GRINM (Guangdong) Institute for Advanced Materials and Technology, Foshan528051, China; Graphene Composite Research Center, College of Chemistry and Environmental Engineering, Shenzhen University, Shenzhen518060, China; Graphene Composite Research Center, College of Chemistry and Environmental Engineering, Shenzhen University, Shenzhen518060, China; Graphene Composite Research Center, College of Chemistry and Environmental Engineering, Shenzhen University, Shenzhen518060, China; Graphene Composite Research Center, College of Chemistry and Environmental Engineering, Shenzhen University, Shenzhen518060, China; Graphene Composite Research Center, College of Chemistry and Environmental Engineering, Shenzhen University, Shenzhen518060, China; Graphene Composite Research Center, College of Chemistry and Environmental Engineering, Shenzhen University, Shenzhen518060, China; School of Materials Science and Engineering, Xiangtan University, Xiangtan411105, China; Department of Mechanical and Materials Engineering, University of Western Ontario, OntarioN6A 5B9, Canada; Eastern Institute for Advanced Study, Eastern Institute of Technology, Ningbo315020, China

**Keywords:** single-crystal NMC, attenuation mechanism, modification strategy, developing route

## Abstract

High energy density and high safety are incompatible with each other in a lithium battery, which challenges today's energy storage and power applications. Ni-rich layered transition metal oxides (NMCs) have been identified as the primary cathode candidate for powering next-generation electric vehicles and have been extensively studied in the last two decades, leading to the fast growth of their market share, including both polycrystalline and single-crystal NMC cathodes. Single-crystal NMCs appear to be superior to polycrystalline NMCs, especially at low Ni content (≤60%). However, Ni-rich single-crystal NMC cathodes experience even faster capacity decay than polycrystalline NMC cathodes, rendering them unsuitable for practical application. Accordingly, this work will systematically review the attenuation mechanism of single-crystal NMCs and generate fresh insights into valuable research pathways. This perspective will provide a direction for the development of Ni-rich single-crystal NMC cathodes.

## Teaser

Utilization of single-crystal Ni-rich NMC cathodes for high energy density lithium batteries poses significant challenges in terms of performances and safety, yet there exists ample room to address the concerns.

## INTRODUCTION

**Figure 1.**
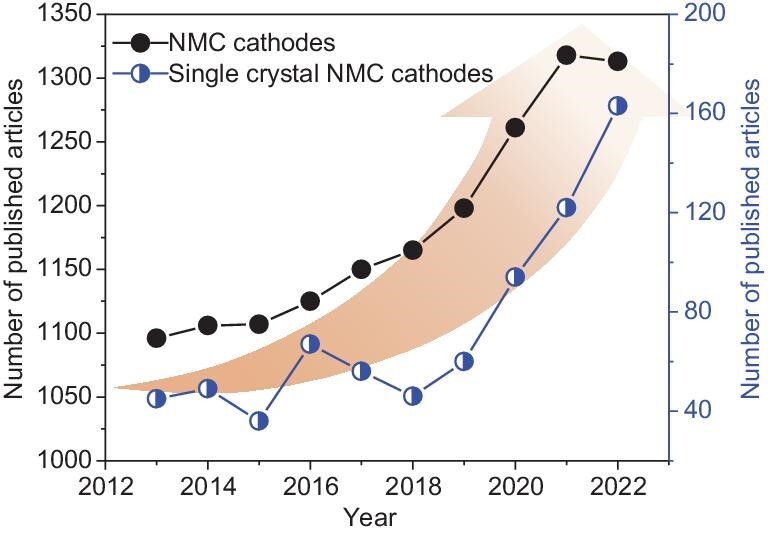
The development trend of NMC-based cathodes over the past 10 years. The collected data are derived from Web of Science from 2013 to 2022, and the corresponding key phrases of NMC cathodes and single-crystal NMC cathodes are ‘NMC (NCM) cathode & lithium battery’ and ‘single crystal NMC (NCM) cathode & lithium battery’, respectively.

What with worldwide reductions in carbon emissions, green electric vehicles (EVs) are experiencing rapid development and are expected to reach 32% market share by 2030 [1], which will ideally be powered by high-energy lithium batteries. The limited specific energy and safety issues of lithium batteries are challenged by the ever-increasing demand of the EV market, leading to the vigorous pursuit of low-cost, high-capacity and high-safety cathodes to enable a long driving range and high-safety lithium batteries. Many families of transition metal oxides and transition metal polyanionic frameworks have been proposed to improve battery energy density [2–6]. Among them, layered transition metal oxides (NMCs) have been extensively studied owing to their high specific capacity (∼278 mAh g^−1^ theoretical capacity) and high working voltage (∼3.8 V) [7–9]. Both polycrystalline NMCs and single-crystal NMCs were developed in order to meet different requirements [10]. In the last 10 years, the amount of literature related to NMC cathodes has increased rapidly (Fig. 1), however, the research scope for single-crystal NMCs is at a nascent stage, and not as wide as that for polycrystalline NMCs.

The polycrystalline NMC contains a clump of nano-sized primary particles, which is conducive to shortening the Li^+^ diffusion pathway and realizing acceptable power density [11,12]. However, owing to its agglomerated structure, it is prone to particle cracking along the grain boundaries among the primary particles, induced by the anisotropic volume variation of the primary particles during electrochemical processes [13–15]. Particle pulverization exposes fresh surfaces to the electrolyte and increases parasitic reactions with the applied electrolyte, accelerating battery performance fading rate and inducing large amounts of gas production [10,16,17]. The above attenuation processes will be fully enhanced in the condition of high Ni content in NMCs, inhibiting their application in high-energy-density lithium batteries. Single-crystal NMC cathodes are free from interparticle boundaries and microcracking during lithiation and delithiation, presenting improved cycling and thermal stability, and have emerged as a promising cathode nowadays [18–21]. In the condition of low nickel content, namely Ni ≤ 60%, the single-crystal NMC demonstrates excellent electrochemical performance. For instance, Dahn's group did a systematic study on single-crystal NMC532, which could maintain thousands of cycles without structure degradation [22–25]. However, when the Ni fraction is above 80% and tested with high cutoff voltages (>4.3 V vs. Li^+^/Li), single-crystal cathodes also present poor cycle performance, even worse than polycrystalline NMC cathodes with the same Ni content [10,26,27]. Hence, for safer practical operation of the high-energy battery system, it is important to clarify the attenuation mechanism of single-crystal Ni-rich NMC cathodes and learn the previous reported modification strategies, then to propose valuable suggestions.

In this review, we will focus on three questions by comparing polycrystalline NMCs and single-crystal NMCs: (i) What drives the faster capacity-attenuation process of Ni-rich single-crystal NMCs compared to polycrystalline NMCs? (ii) Can we find efficient strategies to utterly solve the issues of Ni-rich single-crystal NMC cathodes? (iii) Can single-crystal morphology really break the bottleneck of Ni-rich NMC cathodes? Here, the material synthesis routes and electrochemical performance comparison of polycrystalline NMCs and single-crystal NMCs are firstly summarized and discussed. The differentiation analysis of the attenuation mechanism between single-crystal NMCs and polycrystalline NMCs, especially for Ni-rich components, are also reviewed and discussed. Then, performance optimization strategies for single-crystal NMC cathodes are summarized, including material modification, electrolyte screening and electrode structure design. According to the above summarized capacity decay mechanism and the corresponding optimization strategies, a perspective on how to develop high performance single-crystal NMC cathodes is proposed. This review is expected to inspire further improvement of single-crystal Ni-rich cathodes in the application of high-energy-density lithium batteries.

## NMC CATHODE SYNTHESIS

**Figure 2.**
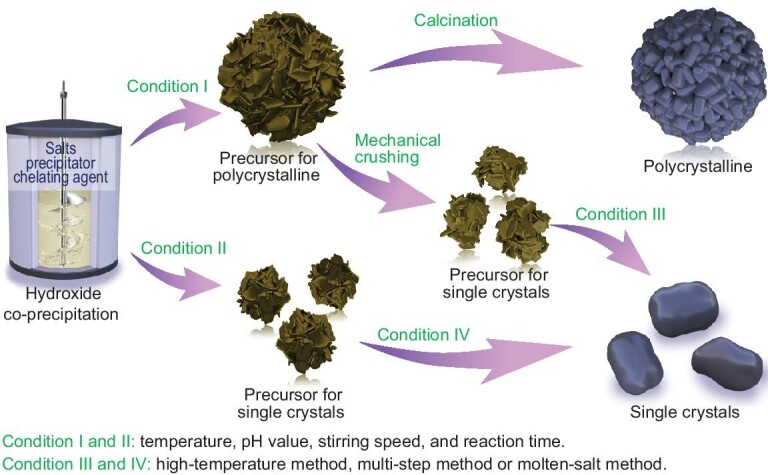
Synthesis routes for polycrystalline NMC and single-crystal NMC cathodes.

The scalable synthesis of single-crystal NMCs is almost in line with polycrystalline NMCs. Methods for different synthetic stages can be permutated and combined with each other. The high-temperature solid reaction combined with co-precipitation has attracted much more attention than other combinations such as sol-gel [28] and spray pyrolysis [29]. According to the difference in calcination conditions, co-precipitation-based synthesis can be categorized into three types: high-temperature synthesis [30], multi-step synthesis [31] and molten-salt synthesis [18]. The co-precipitation method is widely applied to synthesize precursors of both single-crystal and polycrystalline NMCs for practical application [10]. Reactants including transition metal salts, precipitators and chelating agents are dropped continuously into a reactor to obtain hydroxide precipitates under N_2_ atmosphere (Fig. 2). The morphologies, particle size and agglomeration degree of the hydroxide precipitates highly depend on the salt solution concentration, pH value, reaction temperature, reaction time, stirring speed, etc. [32]. Normally, the precursor size for polycrystalline NMC synthesis is larger than that of single-crystal precursors [30]. Typically, radii of the hydroxide precursors are around 10 μm for polycrystalline NMCs and 2–5 μm for single-crystal NMCs [30]. Precursors with medium-sized and dense-fibriform morphology can help to avoid primary particle agglomeration during a high-temperature sintering process to form well-dispersed monocrystalline grains easily [33]. Hence, a precise control of both the primary and secondary particle sizes of hydroxide precursors is very important. Generally, there are two ways to obtain hydroxide precursors of this kind, one is to crumb the large precursors used for polycrystalline NMCs into smaller ones (I and III in Fig. 2), the other is to synthesize precursors of a suitable size directly (II and IV in Fig. 2). A detailed discussion on the advantages and disadvantages of co-precipitation-based high-temperature calcination methods will be carried out in the following paragraph.

High-temperature synthesis is the most simple and common method for commercial mass production of single-crystal NMCs and usually involves three processes: the mixing of precursors and lithium sources, the sintering process, and the optional washing/secondary-sintering process to stabilize the surface of single crystals when it is necessary. Generally, a higher calcination temperature is required to boost the growth of single crystals to accelerate ion migration, while inevitably leading to agglomerated and irregular particles along with increased lithium evaporation and energy consumption [33,34]. Moreover, cationic disordering is aggravated for single crystals under such high temperatures, even generating a rock-salt phase on the particle surface, which increases the impedances and lowers the performance. Sintering temperature and duration, and Li/TM (transition metal) ratios, are dominating factors in high-temperature synthesis, having a significant influence on the crystal structures and particle morphologies of single-crystal NMCs. Li *et al.* [35] conducted a systematic investigation of the Li/TM ratios and sintering temperature of single-crystal NMC622. Increasing the Li/TM (1.05–1.15) ratios and sintering temperatures (925–955^o^C) facilitated the growth of primary particles and promoted the formation of single crystals. For instance, when the temperature was fixed at 925°C, the particle sizes of single-crystal NMC622 were 1–2 μm with Li/TM = 1.05, 3–4 μm with Li/TM = 1.10 and >5 μm with Li/TM = 1.15. According to the lattice constants, the percentage of Ni in the Li layer drops as the amount of excess lithium increases, while the trend is contrary for the sintering temperature. More excess lithium and increased particle size both contribute to a higher electrochemical polarization. Moreover, washed single-crystal NMC622 showed higher capacities than the unwashed ones, but single-crystal NMC622 cycled poorly after the washing–heating treatment due to surface structure damage.

Multi-step synthesis, containing two steps of lithium addition and sintering processes, can conduce to avoid the formation of an impurity phase during single-crystal synthesis at high temperatures. The systematic and comprehensive study of this approach is primarily conducted in LiNi_1-x-y_Co_x_Al_y_O_2_ (NCA) [36]. Typically, Li_5_AlO_4_ impurity has been generated more easily for NCA syntheses with high sintering temperatures and/or high initial Li/TM ratios [37]. Dahn *et al.* [36] synthesized single-crystal NCA with no Li_5_AlO_4_ impurities by the two-step lithiation method. Specifically, the Ni_0.88_Co_0.09_Al_0.03_(OH)_2_ precursor was firstly mixed with LiOH with a Li/TM ratio < 1 (0.8–0.975), then preheated at 485°C and reground, followed by sintering at 485°C, and then calcinated at high temperatures (850–950°C). Additional LiOH was then added to the intermediate to achieve a total Li/TM ratio of 1.01 or 1.02, and calcinated at 735°C. Thus, the Li_5_AlO_4_ impurity phase was successfully prevented for single-crystal NCA synthesis. The capacity retention of as-obtained single-crystal NCA is at least as good as its polycrystalline counterpart for full coin cell cycling. A similar synthesis process was further applied to obtain single-crystal LiNiO_2_ by Dahn's group [38]. For high performance Ni-rich single-crystal NMC cathodes, more investigation needs to be done to optimize the lithium content, sintering temperatures and calcination time in each step.

Molten-salt synthesis, adopting molten salts as fluxes during high-temperature sintering, has been widely applied for single-crystal NMC synthesis, and commonly refers to the solid mixture process, sintering process, washing process and optional secondary sintering process. Fluxes exhibit a molten state at sintering temperature, at which lithium sources and transition metal (TM) sources react with each other to form single crystals. Active elements can dissolve and diffuse in such a molten-salt flux, acquiring additional impetus for fast crystal growth. Only a low sintering temperature is needed to grow single crystals with reduced cationic mixing and particle agglomeration. According to the difference in chemical components, molten salts can be divided into two categories: one is with Li elements (e.g. LiCl, LiNO_3_ and Li_2_SO_4_) and the other is without Li elements (e.g. NaCl and KCl) [39]. The amounts of molten salts are usually several times higher than lithium and TM sources, resulting in large amounts of residual fluxes left on particle surfaces after sintering, which are electrochemically insulated and have to be removed by a washing process. The post-washing treatment makes the particle surfaces more sensitive to the moisture, leading to a significant deterioration of electrochemical performance. An optional secondary sintering process with a lower operation temperature has to be used to repair the sensitive surfaces of single crystals. Additional Li sources are often necessary to reconstruct the particle surfaces without changing the bulk lattice structure. Regular morphologies are achievable by molten-salt synthesis. Zhu *et al.* [35] synthesized a series of (012)-dominated truncated octahedra and polyhedra, (104)-dominated tetradecahedron single-crystal NMCs through molten-salt methods, and (001)-dominated platelets by traditional solid reaction methods. Surface facets were found to play a critical role in the structure stability and cycling performance of single-crystal NMCs. The (012) surface is more reactive than that of (001), which can be evidenced by enhanced surface Ni reduction and higher initial discharge capacity, but poorer cycling stability of truncated octahedron material, as compared to the (001)-dominated platelet. Thus, replacing the reactive (012) facets with less reactive (001) or (104) facets would be effective for high-voltage applications of single-crystal NMCs. Kim *et al.* [40] investigated the influences of NaCl and KCl on morphologies of single-crystal NMC532. The KCl flux promoted the formation of sphere-like single crystals with numerous vague facets, whereas the NaCl flux boosted the formation of octahedron crystals dominated by (101) and (003) planes. However, molten-salt synthesis does well in the synthesis of single-crystal NMCs with regular morphologies. There are still many challenges for molten-salt synthesis such as high costs (e.g. materials, technologies and equipment), a tedious procedure and poor product consistency and production capacity.

## PERFORMANCE COMPARISON BETWEEN SINGLE-CRYSTAL NMCs AND POLYCRYSTALLINE NMCs

Benefiting from their special particle structure and morphology, single-crystal NMC cathodes are generally supposed to show better performance than polycrystalline NMCs in several critical aspects when applied in practical lithium-ion batteries (LIBs), including cycling stability, outgassing and thermal stability. However, the above conclusion remains controversial according to previously published papers, because the electrochemical performance of NMC-based cathodes experiences a disruptive variation at different Ni contents, especially for single-crystal NMCs. So far, there has been no comprehensive electrochemical comparison between polycrystalline and single-crystal NMC cathodes with a variation in Ni content. Therefore, to determine the value of the two materials to a future high-energy-density lithium-battery industry, an accurate performance comparison is essential.

**Figure 3.**
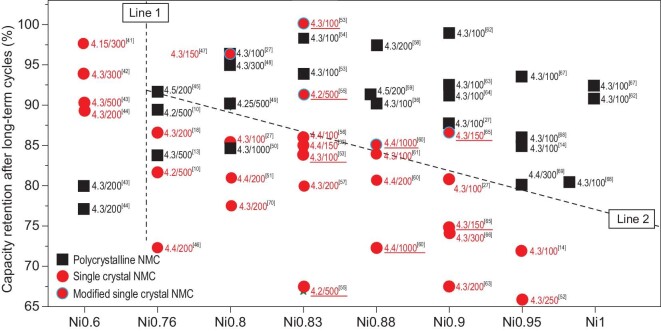
Performance comparison between polycrystalline NMC and single-crystal NMC cathodes. Typically, the icon of 4.15/300 means charging/discharging 300 cycles with an upper limit voltage of 4.15 V.

The comparison of cycling stability between single-crystal NMCs and polycrystalline NMCs with different Ni contents is summarized in [10,13,14,18,27,30,36,41–70]. As can be seen from the summaries, the performance difference highly depends on the Ni content, which can be divided into two regions with a demarcation line between 0.6 and 0.76 (Line 1 in Fig. 3). When the Ni content is <0.6, the cycling stability of single-crystal NMCs is better than for polycrystalline NMCs. The conclusion can be proven by Dahn's work, wherein single-crystal NMC523 was able to undergo many thousands of cycles with minor capacity fade, which is otherwise not possible for polycrystalline NMCs [22,24]. However, when the Ni content is increased from 0.76 to 0.9 (Ni-rich), the result becomes irregular. The overall rough trend is that polycrystalline NMCs show better cycling stability than the un-modified single-crystal ones. However, the performance of single-crystal NMCs can bring a qualitative leap accompanied by different modification strategies (Line 2 in Fig. 3). Sometimes the final cycle capability is even higher than that of polycrystalline NMCs under the condition of Ni-rich components. For example, by synthesis process optimization, Ni-rich single-crystal NMC cathodes present excellent capacity retention after extended cycles—100.09% after 100 cycles at 0.2 C [53] and 96.2% after 150 cycles at 1 C [47]. Material coating, doping and electrolyte additive application also proved useful in improving the stability of the single-crystal NMC cathodes, which will be explained in detail in the following sections. Further increasing the Ni content to 0.95–1.0 (Ni-ultrahigh), polycrystalline NMCs dominate the performance and few single-crystal NMC cathodes are even reported in the literature let alone superior to the polycrystalline particles. However, based on the performance summary in Fig. 3, there is a large space for the electrochemical performance enhancement of single-crystal NMCs. In all, at the condition of Ni-rich components, the electrochemical performance of single-crystal NMCs can be optimized to the level of polycrystalline NMCs or even better. If other properties, including thermal stability after a long-term cycle and overcharge resistance, are better than polycrystalline NMCs, then single-crystal NMCs are worthy of industry selection.

## ATTENUATION MECHANISM COMPARISON OF SINGLE-CRYSTAL NMCs AND POLYCRYSTALLINE NMCs

### Specialty of the attenuation mechanism—intragranular cracks of single-crystal NMCs

**Figure 4.**
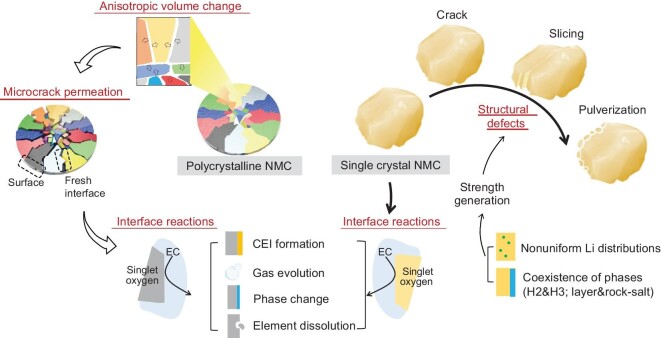
Attenuation mechanism comparison between single-crystal NMCs and polycrystalline NMCs.

As shown in Fig. 4, the anisotropic expansion/shrinkage of the primary particles in polycrystalline NMCs are the driving force for the formation of microcracks, which creates more opportunities for the connection between electrode material and electrolyte and aggravates the side parasitic reactions. Worth mentioning here is that Hu *et al.* [13] provided direct observation and quantification of the lattice and morphological changes of primary particles in polycrystalline NMCs, and further correlated this with the formation and evolution of microcracks/macrocracks and eventually microfractures within the polycrystalline NMC particles.

Single-crystal morphology is designed to solve the severe issues induced by the intergranular cracks in polycrystalline NMC cathodes. However, in practical operation, single-crystal NMC cathodes suffer from slice gliding, intragranular cracks and particle pulverization (Fig. 4), especially under conditions of high voltage, high temperature and high Nickel content [18,27,43,71], and the crack sites are parallel to the particle surface in specific planes or even across the whole particle. A formation mechanism has been proposed and discussed to illustrate the observed issues, but there is no consistent understanding so far. Planar gliding may result in crack formation under the penetration of electrolyte and the following parasitic reactions [72]. As to the prominent intragranular cracks, the possible causes include internal stress between different phases [73], a dislocation-based crack incubation mechanism [74] and oxygen vacancies [75]. Particle surface pulverization was also noticed on single-crystal NMC cathodes after long-term cycling, which can be attributed to the crystal structure mismatch of the surface zone and the non-negligible volume variation [71,73].

**Figure 5.**
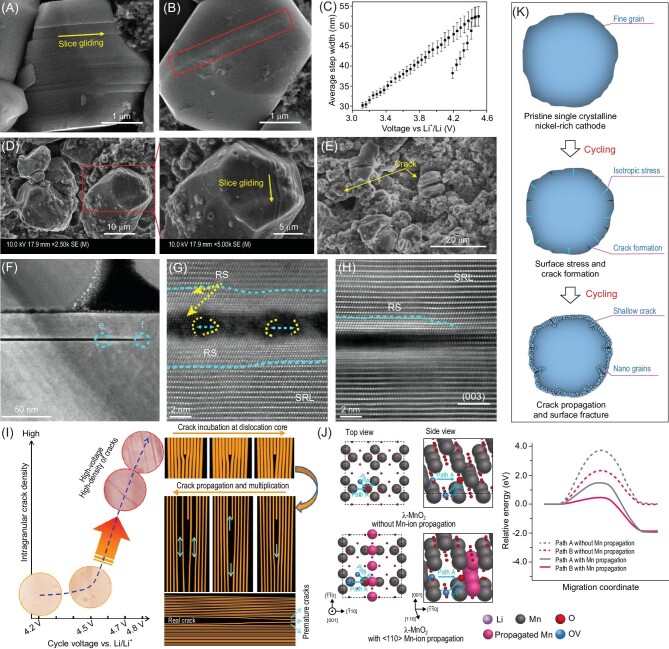
(A and B) Scanning electron microscope (SEM) images of single-crystalline NMC initially charged to 4.8 V (A) and discharged to 2.7 V (B). Adapted with permission from ref. [18]. Copyright 2020 American Association for the Advancement of Science. (C) Quantitative analysis of the observed gliding width changing along the voltage; the increasing and decreasing behavior indicates the reversible gliding process during the charge–discharge process. Adapted with permission from ref. [18]. Copyright 2020 American Association for the Advancement of Science. (D) SEM images of a LiCoO_2_ cathode after 500 cycles, tested in designed electrolyte. The corresponding cut-off voltage is 4.5 V versus graphite. Adapted with permission from ref. [72]. Copyright 2022 Wiley-VCH Verlag. (E) SEM image of a LiCoO_2_ electrode after 100 cycles, tested in carbonate electrolyte. Adapted with permission from ref. [72]. Copyright 2022 Wiley-VCH Verlag. (F–H) An intragranular crack in single-crystal NMC811 after 100 cycles. Adapted with permission from ref. [73]. Copyright 2020 Elsevier. (I) The relationship between intragranular cracking level and cycle voltages; there is almost no crack generated when cycled below 4.5 V, and the intragranular density increases sharply while above 4.5 V. Adapted with permission from ref. [74]. (J) Schematic of oxygen vacancy migration routes with and without Mn-ion propagation in density function theory (DFT) calculations. Adapted with permission from ref. [75]. Copyright 2019 Wiley-VCH Verlag. (K) Schematic illustration of surface nano-grain formation processes in single-crystal NMC cathodes. Adapted with permission from ref. [71]. Copyright 2020 Elsevier.

Bi *et al.* [18] observed lattice gliding in single-crystalline NMC cathodes during the charge process to high voltages beyond 4.3 V, versus graphite (Fig. 5A–C). The high-voltage cycled NMC cathode presented gliding lines along the (003) plane and vertical to the c-axis of the layered structure. *In situ* atomic force microscopy (AFM) measurements have captured continuous morphological changes during electrochemical processes and have proved that the gliding tends to return to its original state during discharge, showing a certain reversibility. Moreover, the high-resolution transmission electron microscope (HRTEM) results confirmed that the layered structure and chemical conditions are well maintained in the gliding places, which excludes the influence of phase change and oxygen loss on the morphology change. Based on the above analysis, the observed slice gliding was attributed to the stress induced by the Li concentration gradient in the lattice during Li^+^ diffusion. Similarly, gliding is also noticed in the LiCoO_2_ system. For instance, Zhang *et al.* [72] built a robust cathode electrolyte interphase by applying an all-fluorinated electrolyte, which successfully protected the reversible planar gliding along the (003) plane by suppressing the element dissolution and electrolyte penetration (Fig. 5D). In contrast, the unprotected LiCoO_2_ cathode experienced serious crack formation during cycling, resulting in worse electrochemical performance (Fig. 5E). Hence, it can be concluded that slice glidings may evolve into intragranular cracks, but it is not the only factor according to the published literature. Generally, strain difference between different phases, the dislocation-based crack incubation mechanism and oxygen loss are the commonly mentioned causes for particle cracks [73–75]. Lin *et al.* [73] carried out an atomic-scale study on the development of intragranular cracks in Ni-rich cathodes, proving that Li/Ni antisite regions with lattice distortion are the nucleation sites for intragranular cracks (Fig. 5F–H). The formed electrochemical inactive rock-salt phase with a different strain to the layered structure, and the formed Coulombic repulsion, are regarded as the two contributors to cracks in the primary particle, which tend to pull apart the rock-salt phase from its original position. However, Ryu *et al.* [27] attributed the apparent structure defects to the inhomogeneous lithium concentration in a single cathode particle, which induces non-uniform stress leading to crack production. The non-uniform stress is not just distributed on the particle surface but also occurs inside the particle because of the local uneven distribution. Different to the strain-induced mechanism, a dislocation-based crack incubation mechanism was proposed by Yan *et al.* [74], suggesting that the intragranular cracks were initiated from the grain interior instead of the grain boundaries or particle surfaces. As shown in Fig. 5I, intragranular cracks can be extensively initiated when the charge voltage exceeds a critical value, such as from 4.5 V to 4.7 V, and the corresponding nucleation sites for crack incubation are located at the dislocation places. During long-term cycling, the formed cracks grow and propagate along the (003) plane, resulting in real cracks. This opens the gap between particle interior and electrolyte leading to the deterioration of cycle performance. Lee *et al.* [75] focused on the influence of oxygen vacancies on particle crack generation, and reported the behavior of oxygen vacancies in single-crystal cathodes during the charge and discharge processes. They prepared a series of single-crystal materials including layered oxides (LiCoO_2_ and Ni-rich cathodes), spinel LiMn_2_O_4_ and Li-rich layered oxide, and noticed that the crack planes of these materials are all related to the {111} plane of cubic close-packed oxygen stacking in the oxygen sublattice, which was regarded as the main contributor to the intragranular crack in single-crystal cathodes. During electrochemical cycling, the existence of oxygen vacancies will induce continuous diffusion of oxygen vacancies and transition metals until a thermodynamically stable state is achieved, by lowering the migration energy (Fig. 5J), resulting in oxygen vacancy condensation and particle cracking along a certain orientation. Single-crystal particle surface pulverization is also observed during cycling, which produces fractured grains on the surface, producing a large amount of fresh surface and causing additional side reactions. Chen *et al.* [71] attributed the surface cracking and pulverization to the different volume change between the particle margin and core of the tested single crystals, which firstly formed shallow cracks and then propagated from the surface towards the core direction, generating nano-grains on the surface as shown in Fig. 5K. By constructing a spinel phase coating layer on the single-crystal surface, the crack generation was significantly reduced and the integrity of the whole particle was well maintained.

Based on the aforementioned analysis, it is evident that there exist diverse forms of morphology evolution during cycling in single-crystal cathodes. Furthermore, even for identical evolutionary processes (such as intragranular cracking), multiple mechanisms have been proposed, thereby adding complexity to the study of this material. In contrast, with regards to polycrystalline NMC cathodes, the mechanism behind performance degradation is well-established and involves strength-induced microcracks followed by subsequent side reactions. Hence, the formulation of performance enhancement measurements is more targeted, such as radically oriented primary particle design and gradient material design. To realize the electrochemical performance enhancement of single-crystal NMC cathodes, more attention should be paid to synthesis processes. The different structure degradation phenomenon can be attributed to the different synthesis conditions, which determine the material crystallinity, crystal plane, particle size and defect degree [21,39].

### Generality of the attenuation mechanism—lattice oxygen release of single-crystal NMCs

The aforementioned microcracks, slice gliding, particle cracks and surface pulverization are associated with performance decay owing to the lack of continuous ion transport channels and the exposed fresh surface, but they are not the intrinsic reasons for its attenuation. The performance decay can be attributed to the release of lattice oxygen and its destabilized behaviors (interface reactions), including cathode–electrolyte interface (CEI) formation, gas evolution, phase change and TM ion dissolution (Fig. 4). The oxygen release processes and their effect will be clarified in the following sections.

### Proof of lattice oxygen generation 

**Figure 6.**
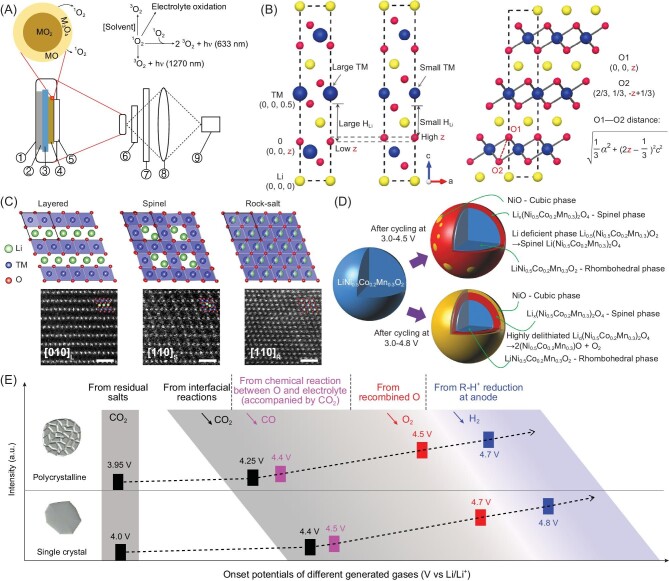
(A) Singlet-oxygen detection technology applied in lithium-ion batteries. Adapted with permission from ref. [79]. Copyright 2018 Elsevier. (B) NMC structure viewed from the [010] direction and the expression of the shortest O–O distance in NMC material. Adapted with permission from ref. [80]. Copyright 2021 American Chemical Society. (C) Phase change of NMC cathodes from layer structure to spinel structure and then to rock-salt structure during electrochemical processes. Adapted with permission from ref. [82]. Copyright 2020 Wiley-VCH Verlag. (D) The attenuation mechanism of an NMC cathode under the condition of high cut-off voltages. Adapted with permission from ref. [83]. Copyright 2013 Wiley-VCH Verlag. (E) Gas production comparison between polycrystalline NMCs and single-crystal NMCs, including gas categories (CO_2_, CO, O_2_ and H_2_) and production onsets. Adapted with permission from ref. [10]. Copyright 2022 Elsevier.

Oxygen release of layered NMC cathodes has been pointed out in the literature by Amine's group [76] and Muto *et al.* [77]. They mention that the observed structural change on the particle surface is accompanied by oxygen loss. However, a direct detection of lattice oxygen release from NMC cathodes is not reported until Hubert's group disclosed their results. They found that the NMC materials charged at high voltage released oxygen at room temperature and the evolution onsets correspond well with the onsets of CO_2_ and CO gases. To support their hypothesis, spinel LiNi_0.43_Mn_1.57_O_4_ was applied and charged to 5 V vs. Li/Li^+^ to prove that oxygen release is the intrinsic reason for CO_2_ and CO evolution from NMC cathodes, because there is no CO_2_ and CO production in this spinel cathode [78]. Although the above result accounts for oxygen release from layered cathodes, there is no direct evidence for the oxygen release. To further explore the oxygen release process, Huber *et al.* [79] directly detected singlet oxygen by monitoring its optical signal. A specifically designed pouch cell was applied to perform the above experiments and was equipped with a quartz glass window for detection (Fig. 6A). Based on this method, singlet oxygen is detected by its characteristic 633-nm luminescence, which happens from both NMC cathodes and over-lithiated NMCs at high degrees of delithiation during the charging process, accompanied by the evolution of CO and CO_2_. Theoretically, Hu *et al.* [80] calculated the important structural parameter of NMC materials—oxygen position, which could be described as 6c (0, 0, *z*) by Wyckoff sites (Fig. 6B). There are two aspects to understand when it comes to the importance of the *z* value, including the lithium diffusion kinetics in the lithium slab [81] and the structural stability of NMC materials at high charge state. By learning the structure information of NMC materials, we found a clear linear relationship between the *z* value and the normalized cation size difference in NMC materials. Based on this, the authors calculated the shortest O–O pair distance (L_O–O_) by the following equation: ${L}_{{\mathrm{O - O}}} = \sqrt {\frac{1}{3}{a}^2 + \ {{( {2z - \frac{1}{3}} )}}^2{c}^2} \ $(a and c come from the position sites of lithium and TM, corresponding to 3a (0, 0, 0) and 3b (0, 0, 0.5)), and the final results indicate that the possible shortest L_O–O_ is 2.49 Å, higher than the O–O dimer (1.45 Å) in standard peroxo species. Therefore, it is impossible to generate O–O from the bulk structure of NMC cathodes, when the layered structure is well maintained. This work appropriately proved that the released oxygen is in the form of singlet oxygen [79], and the detected oxygen gas at ultra-high voltages could be considered the fast combination of singlet oxygen near the particle surface [10].

Layered, spinel and rock-salt phases are frequently observed on the cycled NMC particle surface, owing to their similar TM-O frameworks with close lattice spacing, which can be regarded as further proof of oxygen release. The layered structure presents a hexagonal structure with the $R\bar{3}m{\mathrm{\ }}( {a = 2.877{\mathrm{\ {\mathring{\rm A}}}},{\mathrm{\ b}} = 14.288{\mathrm{\ {\mathring{\rm A}}}}} )$ space group, where lithium, TM and oxygen occupy 3a, 3b and 6c Wyckoff sites, respectively. As for spinel and rock-salt phases, the corresponding space groups are $Fd\bar{3}m( {a = 8.219{\mathrm{\ {\mathring{\rm A}}}}} )$ and$\ Fm\bar{3}m( {a = 4.177{\mathrm{\ {\mathring{\rm A}}}}} )$. The three mentioned atomic structural models and the corresponding high-resolution high-angle ring dark-field image-scanning transmission electron microscopy (HAADF-STEM) images are demonstrated in Fig. 6C, and show that the three phase models have similar unit cells, as marked by the black rhombus [82]. The layered phase change during the electrochemical test can be attributed to the loss of lithium and oxygen, and the corresponding composition on the particle surface goes from LiTMO_2_ to TMO. With an increase in charge cut-off voltage, such as from 4.5 V to 4.8 V, surface structure reconstruction is more serious [83], as illustrated in Fig. 6D. In this process, more oxygen is released from the layered structure and facilitates the interlayer migration of Ni ions during delithiation in NMCs, creating a higher possibility that the phase transition phenomenon will occur. The phase transformation processes from the layered structure to rock-salt were monitored by *in situ* selected-area electron diffraction (SAED) measurements with a transmission electron microscope (TEM), and proved that the rock-salt phase cannot be convertedback to its original state [67].

#### Parasitic reaction between released oxygen and electrolyte

Real-time observation of interfacial reactions has been challenging. It is often complemented by indirect characterizations, including impedance spectra, CEI formation and phase reconstruction. These strategies can demonstrate the existence of interfacial reactions, but the corresponding mechanism and the starting voltage is difficult to determine. Unfortunately, these parameters are extremely important for large-scale market application, which involves battery stability and safety. *In situ* measurements, for instance *in situ* TEM, *in situ* AFM and *in situ* X-ray diffraction (XRD), have undergone rapid development in recent years in order to determine the complex reactions inside the battery. Frequently mentioned phenomena, including surface phase reconstruction [67] and anisotropic volume expansion [13], are all captured by *in situ* techniques. However, these techniques are divorced from the real battery environment. Gas-generation monitoring of battery enabled by *in situ* differential electrochemical mass spectrometry (DEMS) has experienced rapid development, and could accurately obtain the onset voltages of gas production, gas categories and gas contents. This information is conducive to clarifying the mechanism of the interfacial parasitic reactions, because gas generation is the direct feedback of the interface chemical reactions induced by the interaction between electrolyte and released oxygen. Except for gas evolution, the accompanying side reaction results also include CEI formation, phase change and TM dissolution, both in polycrystalline NMCs and single-crystal NMCs (Fig. 4). For instance, Hu *et al*. analyzed the gas production mechanism during whole cycling via *in situ* DEMS, including coin cell and pouch cell, and excluded the possibility that electrochemically driven electrolyte decomposition could produce gases [10]. This work discusses the gas production processes in the reaction between released oxygen and electrolyte, and quantifies and compares the gas content of polycrystalline NMCs and single-crystal NMCs, including CO_2_, CO, O_2_ and H_2_ (Fig. 6E). Single-crystal NMC76 and polycrystalline NMC76 were selected to have contact with the same electrolyte and charged from 4.3 to 4.8 V. During the charging process, four different gases, namely CO_2_, CO, O_2_ and H_2_, were detected in both cases. For polycrystalline NMC76, the initial voltages of CO_2_, CO, O_2_ and H_2_ evolution were 3.95, 4.4, 4.5 and 4.7 V respectively, which were lower than the values tested for single-crystal NMC76. For example, O_2_ did not appear in single-crystal NMC76 until 4.7 V, while it was detectable in polycrystalline NMC76 at 4.5 V. Moreover, the amount of gas detected from single-crystal NMC76 under a high charge voltage was even less than that from polycrystalline NMC76 under a low voltage. It is thus clear that a higher electrochemical driving force is usually required for gas production with single crystals than polycrystals due to the different crystallinity and morphologies [10]. The differences in morphologies, crystallinity and the number of oxygen defects between polycrystals and single crystals may all lead to delayed gas release from single-crystal NMCs. At the same time, it is also observed that the single-crystal NMC also has slower kinetics than the polycrystal NMC at the same current, which may also apply to the delayed oxygen release from the larger single crystals. Hubert's group did a great job on the discussion of battery gas generation mechanisms involved in both the cathode side and anode side. They confirmed the existence of singlet oxygen in layered cathodes. Meanwhile, H_2_ production processes were also illustrated by equipment design [79]. Recently, Li *et al*. used differential scanning calorimetry (DSC) and *in situ* heating high resolution X-ray diffraction-mass spectrometry (HRXRD-MS) technology to determine the reaction position of thermal runaway, and found that the reaction between oxygen and electrolyte was the trigger reaction of thermal runaway [84]. The release of lattice oxygen is necessarily accompanied by the dissolution of TM ions, which influences the CEI composition and thus the surface stability of the cathode. Subsequent migration of TM ions through electrolyte and deposition on the surface of the graphite anode would also lead to an overall electrochemical decline. Eldesoky and Hu *et al*. showed that inhomogeneous TM deposition on the anode is prevalent, and is highly associated with non-uniformity in pressure, current density and the anode surface morphology. In light of the limited solubility of Ni (<0.1%) in the electrolyte for Ni-rich single crystals, it is not expected that the detrimental effects of TM dissolution on capacity retention are primarily due to active material loss. Instead, these effects can be attributed to the migration of TM ions causing the degradation of the cathode's surface CEI and deposition-induced harmful reconstruction of the anode's surface solid electrolyte interface (SEI) [85,86]. Accordingly, for commercial cathode production, surface lattice fixation is imperative and significant.

## PERFORMANCE ENHANCEMENT STRATEGIES

**Figure 7.**
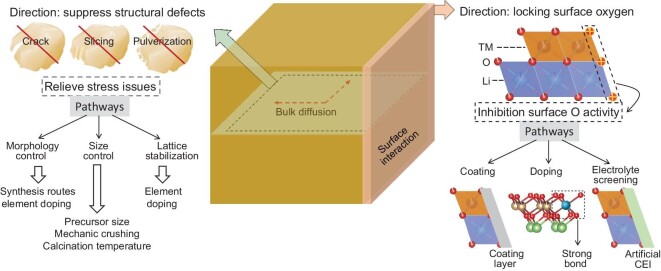
Ni-rich NMC cathode modification strategies, including surface and bulk.

Strategies for the performance enhancement of NMC-based cathodes are mainly carried out in two ways: surface modification and bulk structure binding (Fig. 7). Regarding the material surface, the main purpose of the applied measurements is to stabilize material surface oxygen atoms and inhibit direct contact with electrolyte, and the corresponding strategies include coating layer construction, element doping and electrolyte screening. In terms of lattice stabilization, especially for single-crystal NMC cathodes, the main aim is to prevent the appearance of structural defects in the initial stage of material synthesis or during testing, for instance cracking, slicing and pulverization. Morphology control, particle size control and lattice stabilization (element bulk doping) are the frequently used methods. The above-mentioned strategies will be clarified in detail in the following sections.

### Controllable material preparation

#### Material morphology design

**Figure 8.**
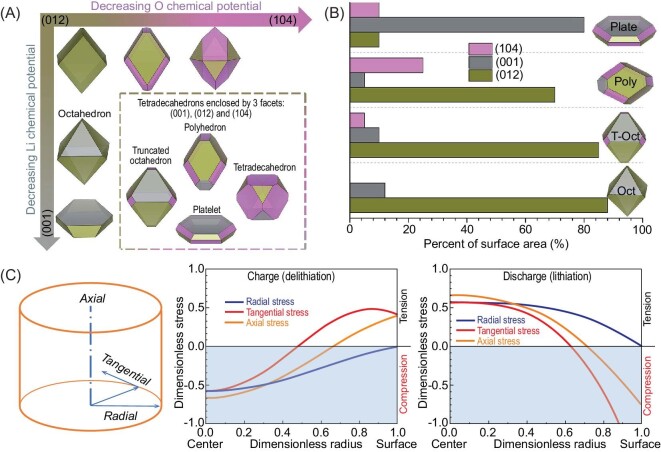
(A) Schematics presenting crystal morphologies as a function of O and Li chemical potentials. The inset shows the possible tetradecahedrons composed of three facets, (001), (012) and (104). Adapted with permission from ref. [35]. Copyright 2019 Royal Society of Chemistry. (B) The relationship between crystal morphologies and crystal facets. Adapted with permission from ref. [35]. Copyright 2019 Royal Society of Chemistry. (C) COMSOL simulation to analyze the mechanical strength of single-crystal NMCs via a cylindrical diffusion-induced-stress model. Adapted with permission from ref. [18]. Copyright 2020 American Association for the Advancement of Science.

The polycrystalline NMC particle is formed by aggregating numerous nano-sized primary particles, which is conducive to shortening the Li-ion diffusion pathway and improving the power density [11]. However, the agglomerated NMC particles experience serious cracking during long-term cycling induced by the anisotropic volume change, which has been regarded as the original driving force for capacity attenuation [13]. Hence, much attention has been devoted to solving the cracking issue, and the main aim is to prepare mesoscale-oriented architecture of the nano-grains, such as a radically oriented primary particle [48,64]. As to the single-crystal NMC particle, it presents a regular shape and has close contact with the electrolyte. Therefore, it is particularly important to optimize the single-crystal plane, especially considering the different activities of the different crystal planes. Chen's group carried out a detailed analysis of the relationship between single-crystal planes and electrochemical performance, and introduced specific strategies for controlling crystal surface growth [35]. They claimed that the (012)-dominated single crystal has worse cycle stability than (001)- and (104)-dominated particles. As presented in Fig. 8A and B, the corresponding crystal structures of (012)-, (001)- and (104)-dominated facets are: truncated octahedron, platelet and polyhedron. If we want to achieve the high stability of single-crystal NMCs, the (001)-dominated platelet morphology is recommended, where the contact area between Li-ion channels and electrolyte is limited. For a high rate capability, the truncated octahedron and polyhedron structures are suggested, because of the existence of numerous open channels to the electrolyte [87]. However, for commercial applications, the surface structure should be modified firstly to enable long-term cycling. With regard to synthesis, we can obtain a low-surface-energy (104)-dominated single crystal by applying a less oxidizing atmosphere or lower reaction temperature [35]. For stable (001)-dominated crystals, more effort should be made to find suitable synthesis conditions.

#### Material size optimization

Generally, single-crystal NMC cathodes present slow Li diffusion kinetics and poor rate capability during electrochemical testing, which can be attributed to the long transmission distance through the micron-sized crystal [88]. In principle, the smaller the particle size, the better the Li kinetics. However, the decreased particle size brings more contact area with electrolyte, inducing the enhancement of interfacial reactions, and leads to a low tap density. As to the single-crystal cathode with large particle size, the corresponding tap and compact densities are high, which is conducive to improving the volumetric energy density, but it causes an increased possibility of cation mixing and decreased Li dynamics. Hence, particle size optimization is very important to balance the comprehensive properties of single-crystal NMCs, including dynamic capability, cycling stability and the volumetric energy density requirement for electrode preparation. The single-crystal particle size can be regulated in different synthesis routes, but the processes of each are different. In the molten-salt synthesis route, regulating the content of molten salts, and the lithium excessive ratio, are the most frequently selected strategies for adjusting particle size. Wang *et al.* [89] successfully synthesized a series of single-crystal NMC cathodes by regulating the LiNO_3_ content, and the corresponding particle sizes range from 0.5 to 2 μm. The particle size growth that comes as a result of increasing the LiNO_3_ content can be ascribed to its low melting point at 253°C and low decomposition temperature at 383°C. Moreover, the single-crystal particle size can also be controllably regulated by changing the calcination temperature in the molten-salt synthesis route, that high temperature favors the growth of single crystals, but the corresponding electrochemical performance experiences severe attenuation owing to the enlarged Li diffusion path and the increased Li/Ni antisite content induced by the high temperature [40]. As to the high-temperature synthesis route, calcination temperature, sintering time and Li content are the main factors affecting the particle size [23,90,91]. Li *et al.* [23] prepared a series of single-crystal cathodes by optimizing Li/TM ratio and calcination temperature, and found that the fastest crystallite growth condition is 970°C and a Li/TM ratio of between 1.2 and 1.25. Overall, what is the optimal single-crystal size for balancing electrochemical performance? Bi *et al.* [18] carried out a detailed COMSOL simulation to illustrate the suitable particle size, and verified that intergranular cracks can be suppressed by reducing the particle size to ∼3.5 μm (Fig. 8C). As to the best size selection, it surely depends on market demand, which will also influence the decision on synthesis conditions such as sintering temperature, time and Li metal.

#### Material coating and doping modification

Electrode material modification is an important and rigorously demonstrated strategy for electrochemical performance enhancement, and has been discussed and summarized in recent published review papers [19,20,39]. Material coating and doping are the two most frequently adopted methods in single-crystal NMC cathodes, and can enable high cycle ability and rate capability [92–94]. However, the two methods have different mechanisms for performance improvement. With regard to the doping method, it is very important to improve the performance of single-crystal NMCs. This mainly works in the following ways: (i) Lower the oxygen atomic activity. The surface oxygen release and the following reaction with electrolyte contribute to material surface reconstruction and gas production, which are the main causes of performance degradation and safety issues [84,95,96]. Introducing transition metal ions that can form strong covalent bonds with oxygen atoms is an effective method to bond oxygen and reduce its reactivity, and the frequently applied transition metal ions include Al, Zr and Nb [93,94]. Guo *et al.* [94] designed a high-valence Nb^5+^ doped single-crystal LiNi_0.83_Co_0.12_Mn_0.05_O_2_ with fewer planar slips and intragranular cracks for cycles under high voltage. The doped Nb^5+^ regulates the grain size of the single-crystal by reducing the surface energies to alleviate intragranular cracks. Apart from the formation of strong covalent Nb-O bonds for lattice stabilization, a heterogeneous structure with Nb^5+^ is constructed on the outer surface, which restrains surface reconstructions and improves interface stability. (ii) Suppress lattice distortion at the high delithiation state. The lithium concentrations in single-crystal NMCs are spatially inhomogeneous during repeated cycling, resulting in the coexistence of multiple phases and non-uniform stress [27]. The uneven bulk strength will generate structural defects, like cracking, slicing or pulverization, eventually contributing to fast performance attenuation. Suitable foreign ion introduction is recommended, such as Mg, Ti or Ta [97–100], which work like a pillar, alleviating lattice distortion during deep charging. (iii) Increase Li-ion diffusion dynamics. The Li-ion transport capacity of the micro-sized single-crystal NMC cathodes is poor, leading to poor rate capability and strong stress in the particle bulk, so it is necessary to improve Li-ion kinetics via appropriate methods. The doping of specific elements can enable larger layer spacing along the c-axis, thus reducing the diffusion energy barrier of Li-ion and achieving structural stability. This has been verified by using W, B, Ta, F, etc. [62,101–103]. Double- or multi-element doping has also been widely adopted to achieve more of the aforementioned advantages at the same time [104]. For instance, Zhang *et al.* [50] introduced four kinds of elements (Ti, Mg, Nb and Mo) into the Ni-rich cathode, which realized zero volumetric change during de-/lithiation and high capacity simultaneously. At the same time, the material thermal stability was improved. Ou *et al.* [93] reported an Al/Zr co-doped single-crystal LiNi_0.88_Co_0.09_Mn_0.03_O_2_ to circumvent the instability issue, and found that more soluble Al ions are adequately incorporated in the lattice while the less soluble Zr ions are prone to aggregate in the outer surface layer. The synergistic effect of Al/Zr co-doping in the NMC lattice improved Li-ion mobility, relieved the internal strain and suppressed Li/Ni cation mixing upon cycling at high cut-off voltage.

Surface coating/modification is another important and effective strategy to suppress the performance decay issue. The main working mechanism of this surface engineering includes [19,60,105–107]: (i) buffer layer construction to suppress interfacial side reactions between electrode material and electrolyte; (ii) decreasing interface activation energy by coating ionic/electronic conductors; (iii) introducing foreign ions to bond surface lattice oxygen during the coating process. By coated-layer construction, the unwanted interfacial parasitic reactions will be highly suppressed, and the layer structure will be maintained. Zhang *et al.* [108] found that the surface Ni-rich rock-salt phase plays a major role in the cycle stability of single-crystal NMC. And a post-sintering treatment with additional Li source under a relatively low temperature can make the surface Ni-rich rock-salt phase restore itself to the stable layered phase, which can significantly enhance the cyclic performance. Moreover, with a high ionic/electronic conductive coating layer, the interface charge transfer impedance will be decreased. Zheng *et al.* [92] revealed that Li_1.8_Sc_0.8_Ti_1.2_(PO_4_)_3_ (LSTP) surface modification can help to construct a robust interphase between single-crystal LiNi_0.6_Co_0.1_Mn_0.3_O_2_ and the electrolyte, which can prevent NMC corrosion by electrolyte, and the stability of the mechanics can improve the intergranular cracks caused by long cycles under harsh conditions. Moreover, the LSTP conductive modification layer can enhance rate capability by facilitating Li^+^ transport. However, it is not easy to achieve a completely uniform and dense coating layer [109]. Moreover, especially for single-crystal materials, the variation of particle volume during repeated cycling processes will lead to the rupture of the coating layer and reduce its function. Therefore, to some extent, the coating modification can alleviate the attenuation process of materials, but it cannot solve the problem completely. Therefore, it is suggested that along with achieving the uniformity of coating to the maximum extent, other modification techniques such as doping should also be adopted to improve the comprehensive properties of the applied single-crystal materials.

#### Electrolyte optimization

**Figure 9.**
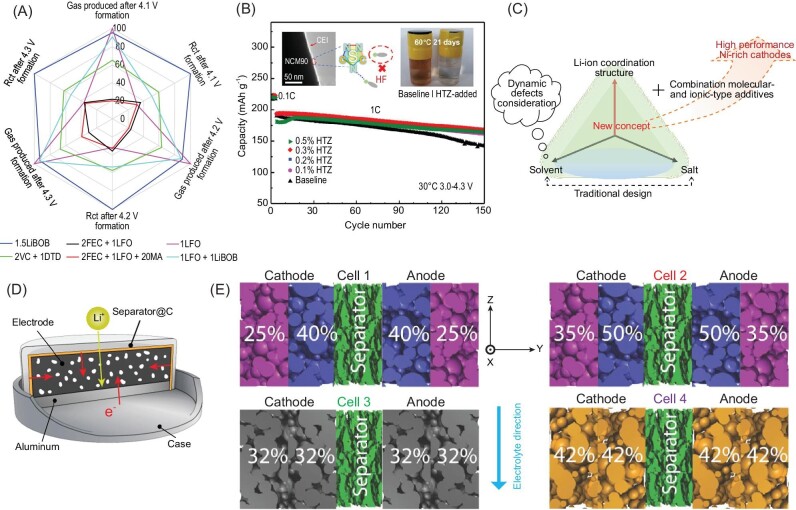
(A) Radar plot of single-crystal NMC811/graphite pouch cell to compare the gas evolution content and impedance formation in different electrolyte environments. Adapted with permission from ref. [22]. (B) Electrolyte additive effect on the electrochemical performance of LiNi_0.9_Co_0.05_Mn_0.05_O_2_ (NCM90). Adapted with permission from ref. [65]. Copyright 2021 American Chemical Society. (C) New electrolyte design route of single-crystal NMC cathodes, including traditional solvent and salt optimization, Li-ion coordination structure and multi-type additive application principles. (D) Three-dimensional conductive network construction by conductive substance coating on the separator, eliminating the influence of electronic conductance on the rate capability of the thick electrode. (E) Schematic illustration of electrode structure design. The inset white numbers are the electrode porosities. Adapted with permission from ref. [114].

Interface side reactions and structural defect formation of single-crystal NMC cathodes can lead to severe performance degradation over long cycles, especially at high voltages and/or high temperatures (Fig. 4). Proper electrolyte application can suppress such interface side reactions and TM dissolution by the formation of a protective CEI layer, although the screening of electrolyte on single-crystal NMC cathodes is limited compared to polycrystalline NMC cathodes and single-crystal LiCoO_2_. Electrolyte additives are most important and can be divided into two types, molecular and ionic [110]. The selection of molecular additives should consider the relationship between the performance and frontier orbital energy; the higher the highest occupied molecular orbital (HOMO) of the additive the better the oxidation ability, and the lower the lowest unoccupied molecular orbital (LUMO) the better the reducibility. Overall, the principle of molecular additive is that its decomposition reaction should occur more easily than the used solvents, and the sacrifice of additives can effectively stabilize the interface of single-crystal NMCs and electrolytes. Ionic additives usually refer to the Li salts, and are conducive to forming a protective layer by the decomposition of salt anions. Combinations of different-molecular-type and/or different-ionic-type additives are highly recommended for stable interfacial protective layer construction. Dahn's group carried out a comprehensive study on single-crystal NMC electrolytes from low nickel content to Ni-rich cathodes. They found a series of additives that work well for improving the electrochemical performance of single-crystal NMC cathodes, including LiPO_2_F_2_ (LFO) [22], prop-1-ene-1,3-sultone (PES) [23], tris (trimethylsilyl) phosphite (TTSPi) [23] and 1,2,6-oxadithiane 2,2,6,6-tetraoxide (ODTO) [24], as well as their mixture with vinylene carbonate (VC) and fluoroethylene carbonate (FEC). A single-crystal NMC811/graphite pouch cell was selected for the comparison of gas production and charge transfer impedance of the electrolytes with different additives. The radar plot shown in Fig. 9A proves that the combination of FEC (molecular additives) and LFO (ionic additives) has the best overall performance [22].

The electrolyte optimization strategies of Ni-rich single-crystal NMC cathodes should not only consider interfacial stability, but also consider rate capability, through electrolyte design, to enhance the kinetics of the micro-sized single crystals (Fig. 9C). The formed CEI protective layers usually hinder the diffusion of lithium ions and/or charge transfer, resulting in the worse performance of single-crystal NMCs at elevated current density. It is thus quite important to make a trade-off. Zou *et al.* [65] applied 1,2,4–1H-Triazole (HTZ) as electrolyte additive to improve the interfacial stability of Ni-rich single-crystal NMCs. This exhibited a higher HOMO than solvents and promoted the formation of a compact and dense nitrogen-containing CEI layer that effectively alleviated electrolyte oxidative decomposition and boosted both cycle stability and rate capability (Fig. 9B). For electrolyte optimization of Ni-rich single-crystal materials, the regulation of solvent and salt is as important as the selection of additives. As to the solvent selection, the coordination of ethylene carbonate (EC) molecules with Li-ions (in the form of Li^+^(EC)_4_ clusters) should be fully considered, and the coordination number of EC/Li-ion is supposed to be controlled below four to suppress EC decomposition on the surface of high oxidation cathode materials that could cause a high interface impedance [111]. For electrolyte, the lower the viscosity of the solvent, the higher the Li-ion diffusion ability will be, thus dimethyl carbonate (DMC) is a promising candidate owing to its viscosity advantage compared to other carbonate solvents. However, more work needs to be done to determine the optimal solvent ratios, especially when high-temperature and high voltage conditions are considered. LiPF_6_ is a traditional lithium salt of commercial electrolyte that easily experiences decomposition and forms HF during battery operation, resulting in further performance attenuation of single crystals. Thus, more attempts to partly or wholly replace LiPF_6_ with other lithium salts (e.g. LiDFOB, LiBF_4_) are encouraged to achieve better battery performance [112].

#### Battery structure design

The material-level modification of NMC cathodes is of great significance and can fundamentally solve or suppress the intrinsic defects. However, when a battery is prepared, especially under the condition of high loading content for the electrode, new influencing factors will be introduced, and these factors will be the decisive step in terms of the performance. Accordingly, it is urgent and important to summarize the possible influences of electrode structure on electrochemical performance, then to guide researchers to design electrodes with excellent performance. Under the variation of electrode thickness from thin to thick, two important factors will be introduced: electrical and ionic conductivities of the prepared electrode [113]. For instance, the un-calendared thick electrode has a high porosity enabling fast ionic diffusion from the top of the electrode to the bottom, but the corresponding electronic conductivity is limited owing to the loose electrode structure, which contributes to the poor rate performance. The limited electronic conductivity can be solved by building a 3D conductive network as shown in Fig. 9D, where the separator is coated by a conducive substance to enable electron transport from electrode surface to the current collector side [113]. As to the calendared thick electrode, the active particles contact well with the conductive carbon, resulting in superior electrical conductivity. However, the existing ionic diffusion channels are blocked, resulting in poor lithium dynamics in the electrode level, which is more obvious at high current densities. Based on these phenomena, it is necessary to establish the electrolyte maintaining ability on the electrode surface and to infiltrate the electrode adequately before the electrochemical test. Recently, the application of electrode architectures with layers of different porosities has garnered more and more attention. This can tune the wettability of the thick electrodes [114,115]. Normally, there are two tools to fabricate electrode architectures, including the distribution of electrode porosities in different electrode thickness and particle size distribution of the electrode material. Abbos *et al*. studied the relationship between porosity, porosity distribution, particle size distribution and electrolyte infiltration ability via the Lattice Boltzmann Method, showing that electrolyte infiltration can be optimized by electrode architecture design [114] (Fig. 9E). The preparation of thick electrodes and super thick electrodes is the inevitable choice for high-energy-density lithium batteries. The preparation methods can also be optimized through approaches such as dry coating and wet coating [116]. In terms of cost and super-high loading requirement, dry electrode preparation is more advantageous, but the optimization of electrode quality and the relationship between electrode structure and electrochemical performance need to be further discussed.

## CONCLUSION AND PERSPECTIVE

**Figure 10.**
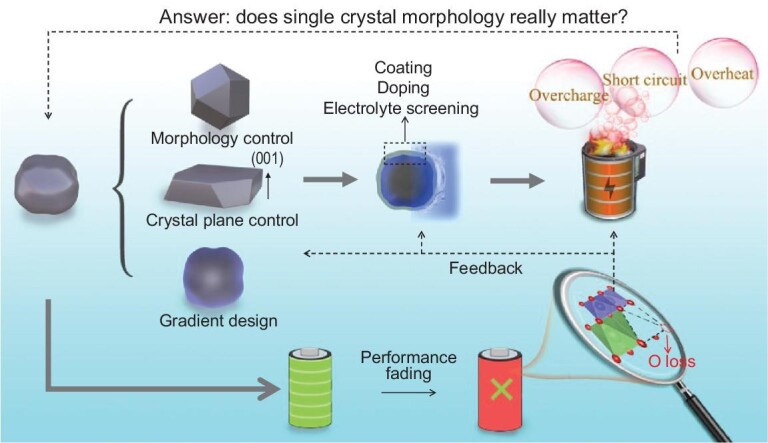
Schematic illustration of the development route for practical single-crystal NMC cathodes.

Considering the growing demand for long-life lithium batteries, the market share of layered NMC cathodes will increase and Ni-rich components will dominate. However, the existing issues of polycrystalline Ni-rich NMC cathodes are difficult to eliminate, and this is now regarded as a bottleneck. Single-crystal NMC cathodes with high thermal stability, low air sensitivity, low gas production content, etc., have garnered attention. However, at high Ni content, the cycle ability of single-crystal NMCs is worse than that of polycrystalline NMCs, and their application is therefore limited. Except for similar attenuation mechanisms, including interfacial parasitic reactions, surface oxygen release and transition metal dissolution, single-crystal Ni-rich cathodes also face dramatic stress variation during repeat cycling, leading to particle cracking, slicing and pulverization. The corresponding solutions have been comprehensively reviewed, including material synthesis (morphology optimization, size control, coating and doping), electrolyte screening and electrode construction, which work to solve the decay issues. But the existing problems of single-crystal Ni-rich NMC cathodes have not been fully solved. Hence, the root causes of the performance attenuation of single-crystal Ni-rich NMC cathodes need to be further explored, and corresponding strategies can be formulated to entirely resolve them. Based on the present situation, we put forward the following suggestions for the development of single-crystal Ni-rich NMC cathodes (Fig. 10):

- **Controllable material preparation**. Single-crystal morphology and size have a great influence on electrochemical performance, so the two factors should be strictly controlled. As reviewed in this work, the related conditions for the particle size and morphology include precursor type, synthesis route (high-temperature, multi-step, molten-salt) and synthesis conditions (temperature, lithium source content, etc.), which should be systematically and comprehensively studied for controllable single-crystal material preparation. Gradient material design has been adopted in polycrystalline NMC cathodes, which suppresses side reactions and achieves a good electrochemical performance. This concept can also be applied to single-crystal particles by using a suitable gradient precursor (low nickel on the surface, Ni-rich in the particle center) to relieve the surface pressure of single-crystal materials.
- **Attenuation mechanism study**. At present, the attenuation mechanism study of single-crystal NMC cathodes is focused on the specific product; the single crystal is synthesized under a particular condition. Different groups get different decay causes, and the parallel comparison is lacking. Through a comparative analysis of the large amount of literature, we can conclude that the interfacial parasitic reactions and the generated bulk strength during delithiation/lithiation are the fundamental reasons for performance attenuation. But the current understanding is not enough to guide material modification and suggest a specific strategy. Therefore, it is suggested that we establish the relationship between precursor characterization, synthesis route, morphology, size and performance attenuation, and then entrench the connection between material synthesis conditions (temperature, lithium content, etc.) and the final performance. An understanding of the relationship between synthesis procedures and material properties is conducive to improving the efficiency of solving the performance decay issues.
- **Advanced characterization techniques**. Advanced methods enabling high resolution and *in situ* tests should be developed to clarify the intrinsic mechanism of performance fading. Presently, Bragg coherent X-ray diffraction imaging (BCDI) [117], transmission X-ray microscope (TXM) tomography [50], atomic-resolution scanning transmission electron microscopy (STEM) imaging [67], *in situ* XRD [27], *in situ* DEMS [10], etc. have been applied in layered cathodes, and can monitor the concurrent appearance of both compressive and tensile strain, and realize three-dimensional reconstructions of the target material during electrochemical processes. Accordingly, it is recommended that these techniques and novel equipment are combined to achieve a description of the fading mechanism in single-crystal NMC cathodes.
- **Application of multiple modification measures**. Some modification strategies work on the performance enhancement of single-crystal NMC cathodes, for instance, coating, doping and electrolyte additive application. However, none of them have the ability to suppress performance fading totally. Based on the principle that the potential value of one plus one is greater than two, the combined action of multiple modification strategies is an important scheme to improve the performance of single-crystal NMC cathodes. Basically, material doping and coating should be carried out for the suppression of structural degradation and the improvement of mechanical integrity during repeated cycling. Moreover, the design and development of high ionic conductivity and high stability electrolyte also need to be applied meanwhile, to suppress the interfacial reaction pressure of single-crystal materials.
- **Does single-crystal morphology matter?** For the large-scale application of single-crystal NMC cathodes, all possible phenomena, including safety issues, should be considered and studied in advance. It is well known that single crystals have a low specific surface area, resulting from suppressed parasitic reactions, which is considered to be one of the main factors that is superior to polycrystalline materials. However, under extreme conditions, for instance overcharging and overheating, do single-crystal NMC cathodes have the same advantages as under normal conditions? For example, particle cracks will occur in the overcharged state, which exposes more fresh surfaces and may cause rapid interface reactions and gas production inside the battery, resulting in battery failure and safety issues. Moreover, the thermal stability of single-crystal NMC cathodes after long-term cycles also needs further study, including the charge and discharge states, to determine the possibility for long-term use as electrode material in power batteries. With the above research and discussion, the posed question in the introduction will be solved and we can truly give an answer as to whether single-crystal NMC cathodes, especially for Ni-rich components, can be applied for next-generation high-energy-density lithium batteries.
